# Mass administration of medicines in changing contexts: Acceptability, adaptability and community directed approaches in Kaduna and Ogun States, Nigeria

**DOI:** 10.1371/journal.pntd.0008857

**Published:** 2020-11-25

**Authors:** Oluwatosin Adekeye, Kim Ozano, Ruth Dixon, Elisabeth Osim Elhassan, Luret Lar, Elena Schmidt, Sunday Isiyaku, Okefu Okoko, Rachael Thomson, Sally Theobald, Laura Dean

**Affiliations:** 1 Sightsavers, Nigeria Country Office, Kaduna State, Nigeria; 2 Department of International Public Health, Liverpool School of Tropical Medicine, Pembroke Place, Liverpool, United Kingdom; 3 Sightsavers, Research Team, Haywards Heath, United Kingdom; 4 Neglected Tropical Disease Programme, Federal Ministry of Health, Government of Nigeria, Abuja, Nigeria; 5 Department of Parasitology, Liverpool School of Tropical Medicine, Pembroke Place, Liverpool, United Kingdom; Instituto de Ciências Biológicas, Universidade Federal de Minas Gerais, BRAZIL

## Abstract

Nigeria has the highest burden of NTDs in sub-Saharan Africa. Commitments to reach the control and elimination of many Neglected Tropical Diseases (NTDs), particularly those amenable to preventive chemotherapy (onchocerciasis, schistosomiasis, soil transmitted helminths, lymphatic filariasis and trachoma) by 2020 are detailed in the London declaration. Strategies to reach targets build on existing approaches, one of which is the use of community directed intervention (CDI) methods to deliver the mass administration of medicines (MAM). However, treatment using this approach has been inconsistent and there are questions about the acceptability and adaptability of these interventions during periods of programmatic, social, and political change. This paper explores the current strengths and weaknesses of CDI approaches in MAM delivery. We consider the acceptability and adaptability of existing MAM approaches to ensure equity in access to essential treatments. Using qualitative methods, we explore implementer perspectives of MAM delivery. We purposively selected programme implementers to ensure good programmatic knowledge and representation from the different levels of health governance in Nigeria. Data collection took place across two States (Kaduna and Ogun). Our results indicate that CDI approaches have underpinned many historic successes in NTD programme acceptance in Nigeria, specifically in Kaduna and Ogun State. However, our results also show that in some contexts, factors that underpin the success of CDI have become disrupted presenting new challenges for programme implementers. Capturing the tacit knowledge of health implementers at varying levels of the health system, we present the current and changing context of MAM delivery in Kaduna and Ogun States and consolidate a platform of evidence to guide future programme delivery and research studies. We situate our findings within the broader NTD literature, specifically, in identifying how our findings align to existing reviews focused on factors that shape individual acceptance of MAM.

## Author summary

Nigeria has the highest number of people affected by neglected tropical diseases in sub-Saharan Africa. Many of these diseases (onchocerciasis, lymphatic filariasis, schistosomiasis and soil transmitted helminths) can be prevented and treated using mass administration of medicines (MAM), which involves giving everyone in communities medicines based on their height. MAM has been going on for a long time in Nigeria and has achieved many successes, however, the programmatic, social, political and economic context in Nigeria is rapidly changing and there is limited evidence about the ability of current MAM approaches to ensure everyone who needs these medicines is able to access them during this time of change. We asked MAM programme implementers at different levels of the health system in Nigeria to tell us what they thought the key strengths and weaknesses of the current programme were and what they would do to make it better to ensure that everyone who needs the medicines would still be able to access them. They told us that the commitment of health workers and community leaders to telling communities about the medicines and encouraging them to accept them as well as the knowledge of the disability these diseases can cause had supported programme success. However, they also told us that in some areas, such as those with security concerns or where communities feel fatigued by the programme, the way communities respond is changing. To address the change in community responses our study participants felt that we need to support programme implementers with the time, tools and resources to build stronger more trusting relationships with communities to ensure everyone can access medicines.

## Introduction

Neglected tropical diseases (NTDs) affect the most impoverished and marginalised populations in the world [1]. Over the last three decades NTDs have received considerable attention and funding, with most recent efforts being targeted toward the attainment of commitments detailed in the London Declaration that pursue the control and elimination of selected NTDs by 2020 [1]. Strategies to reach the targets build on existing approaches, one of which is the use of community directed intervention (CDI) methods to deliver the mass administration of medicines (MAM), i.e. distribution of freely donated medicines at scale, to tackle five of the world’s most prevalent NTDs: lymphatic filariasis (LF), onchocerciasis, schistosomiasis, soil transmitted helminths (STH) and trachoma [2]. MAM has become the primary public health intervention aimed at reaching control and elimination of these diseases [3] and such approaches have been largely successful in reaching those in need. However, treatment has been inconsistent and there are questions about the sustainability of these intervention successes during periods of programmatic, social, and political change [4,5]. As we have reached 2020, it is critical that we evaluate the successes and challenges of implementing MAM to ensure the equitable attainment of the ‘end game’ in diverse and rapidly changing settings [6].

CDI approaches originated in 1995 from the African Programme for Onchocerciasis Control (APOC), which emphasised community engagement as a central component of MAM and established a clear process for community directed treatment with ivermectin [7]. The process relied on sensitisation and mobilisation of community members to select a volunteer community directed distributor (CDD), who the community would support in kind (and/ or cash) to deliver medicines house to house, to every eligible community member [7]. Since inception, this approach has been used in many settings globally and is the key delivery mechanism for MAM in trachoma, LF, onchocerciasis and community-based STH and schistosomiasis programmes [3].

Nigeria has the largest burden of preventive chemotherapy neglected tropical diseases (PC -NTDs) in sub-Saharan Africa [1]. A large proportion of this burden is due to the five most prevalent NTDs targeted by MAM. Therefore, CDI approaches have become a standard model of delivery for preventative chemotherapy in Nigeria [8]. Nigeria was one of the pilot sites for APOC and has a long history of CDI for NTDs. In recent years, scale-up of MAM has been supported nationally across all target States [8]. This provides a good opportunity to study how NTD programmes can adapt to meet the needs of culturally and religiously diverse populations, some of whom live in fragile or security compromised areas, 50.2% live in urban areas and a further 6.8% are nomads [8–10]. The country operates a federal system of government that the NTD program also adopts. The Federal Ministry of Health (FMoH) is responsible for supervision and training of the state and this is cascaded to the Local Government, the front-line health facility (FLHF) staff and then the CDD [11]. The country has 36 states and 774 local government areas (LGAs) within those states which are diverse epidemiologically and programmatically.

This paper draws on the findings of implementation research in Nigeria, conducted with funding from the COU**NTD**OWN research consortium supported by the UK Department for International Development (DFID). The aim of this study was to explore current strengths and weaknesses of CDI approaches in MAM delivery and provide recommendations of good practices and implications for future research. Specifically, we consider the importance of using CDI principles for effective co-ordination of MAM activities and consider the challenges of implementing these strategies in large, diverse programmes over an extended period.

## Materials and methods

### Ethics statement

The study was approved by the Ethics Boards of the Liverpool School of Tropical Medicine (Reference: 15.043RSa) and the Nigerian Federal Ministry of Health (Reference: NHREC/01/01/2007-13/01/2017). All participants were informed about the purpose of the study and provided written informed consent.

### Study setting

In this study, we used qualitative methods to capture the tacit knowledge of programme implementers at varying levels of the health system to present the current and emerging context of MAM delivery in Kaduna and Ogun states.

Data for the study was collected at national level and from two states, Kaduna (north-west) and Ogun (south-west). Their selection helped to achieve maximum variation in context and to understand how a difference in implementation and funding options influence the NTD programme in both states. The states represent maximum diversity due to differing cultural composition, with Kaduna consisting of a mainly Hausa population, and Ogun having a mainly Yoruba population. Typically, Ogun is also viewed as a more ‘secure’ state than Kaduna, with Kaduna experiencing periods of religious fraction and ethnic violence. Both Kaduna and Ogun states are endemic for four of the five PC NTDs, onchocerciasis, lymphatic filariasis, soil transmitted helminths and schistosomiasis. Community directed treatment for PC NTDs has been ongoing for 20 years in Kaduna and 18 years in Ogun [11]. The NTD programme in Kaduna receives significant funding from the UK Department for International Development (DFID) and technical support from the non-governmental organisation (NGO) Sightsavers, whilst Ogun State receives limited funding from the United Nations Children’s Fund (UNICEF) and the World Health Organisation (WHO) on an ad-hoc basis. Ogun state received no technical support from NGOs as at the time of this study.

### Sampling

Within each State, three LGAs were purposively selected to ensure maximum variation in disease prevalence, programme impact (measured by geographic and therapeutic treatment coverage), culture and geography. In Kaduna, one (State) with a border LGA, one northern LGA, and one southern LGA were selected. In Ogun, one central LGA, one eastern LGA, and one border (Country) LGA were selected. Variation in urban/rural LGAs was also aimed for across the sample. Characteristics of each LGA are shown in Table 1.

**Table 1:** Selected LGAs.

States	LGA Selected	Reported therapeutic coverage of community-based MAM (2016) (%)			
		Onchocerciasis^1^	Lymphatic^1^ Filariasis/Soil Transmitted Helminths	Schistosomiasis (School Based)	Urban/Rural (based on programmatic classification of these areas using population density)
Kaduna[12]	Ikara	80	80	N/A	Rural
	Igabi	76	76	99	Urban
	Kachia	78	78	94	Urban
Ogun[12]	Yewa North	78	78	68	Rural
	Ijebu East	54	54	57	Rural
	Abeokuta North	69^2^	40^2^	31	Mixed

^1^ Co delivered through the same platform.

^2^ These figures are slightly different due to variance in the target population of the intervention in this LGA. Those in urban sections of this LGA do not receive onchocerciasis treatment.

### Study design and participants

Key informant interviews were conducted with programme implementers at different levels of the health system (national/Federal, State, and LGA). Participatory stakeholder meetings were also conducted with LGA health implementers, health facility staff, CDDs and teachers. A qualitative approach was used to allow stakeholders at all levels of the NTD programme to share their experiences. The inclusion of stakeholders at all health systems levels supports to address the top down approach to health and intervention design in Nigeria. Key informant interviews took a semi-structured approach and covered a range of topics including successes and challenges of programme implementation and recommendations for strengthening community engagement. Stakeholder meetings with key implementers drew on participatory methods where participants considered and discussed implementation strengths, weaknesses, challenges, and recommendations for improvement.

Purposive sampling was used to select participants for both methods, ensuring good programmatic knowledge and representation from the different levels of health governance in Nigeria. Key informants included NTD programme managers, programme co-ordinators, desk-deworming officers and representatives from civil society. Forty-two key informant interviews were completed as shown in Table 2. CDDs, teachers and health facility staff were also purposively selected from a sample frame of these cadres in each LGA. Other characteristics, such as length of service to the programme, sex and age were also considered. Twelve participatory workshops were completed, one per cadre in each selected LGA. For a breakdown of participatory workshop participants please see Table 3.

**Table 2:** Key informant interview participants.

	Kaduna	Ogun
International NGDO Partner	5	
Federal Ministry of Health	5	
State Ministry of Health	4	4
Local Government Authority (LGA)	9	15
Total	42	

**Table 3:** Participatory workshop study participants.

State	LGAs	Participatory Workshop Participants by Gender					
		FLHF staff workshop		CDD workshop		Teacher workshop	
		Male	Female	Male	Female	Male	Female
Kaduna	Kachia	3	12	6	6	12	3
	Igabi	13	2	14	1	14	0
	Ikara	9	6	14	1	15	0
Ogun	Abeokuta North	0	15	10	4	6	9
	Ijebu East	0	15	6	9	4	11
	Yewa North	7	8	10	3	4	11
Total		32	58	60	24	55	34

### Data collection and analysis

Data collection took place between January and March 2017 by three data collection teams who had been trained for one-week. Study instruments were piloted and modified. Iterative modifications to the data collection tools were also made during the data collection period to ensure the study addresses all research questions. Each interview took approximately one hour, and workshops were approximately 2 hours.

All State data was collected and audio recorded in the local languages, Hausa (Kaduna) and Yoruba (Ogun); and Federal interviews were conducted in English. Data collection took place in a location of participants choosing that was deemed safe for both researcher and participant. Interviews were transcribed verbatim and translated to English, where necessary, a sample were back translated, and recordings were checked for accuracy. Records and notes from observations at the workshops were typed to supplement the analysis. Data was analysed thematically using Atlas.ti (version 6) software. Each of the research team leads led the analysis process with support from the UK research team and in collaboration with national NTD programme partners. The framework approach was used to analyse the data [13].

## Results

Our results can be broadly split into two thematic areas; acceptability and adaptability. Key components of CDI approaches such as the critical role of community leaders in engaging communities for MAM; and the fundamental role of the health workforce in sensitisation and mobilisation, were described by participants as vital components of programme delivery that are continuing to shape medicine acceptance amongst community members. Beliefs and perceptions about medicines and their side effects were also found to be critical in shaping acceptance. The ability of CDI approaches to adapt to variations in socio-political circumstance due to issues such as insecurity, urbanisation, migration, and changing development priorities also emerged as key issues impacting programme success. Finally, adaptability at the micro-level in relation to gender and livelihoods also influenced the ability of certain population groups to access medicines. Each of these areas are discussed within the subsequent results section.

### Acceptability

#### Community leaders remain critical in community engagement for MAM

Some CDDs and teachers, and most FLHF staff, described that community leaders and associated structures such as religious groups, village health committees and market associations were critical in shaping how communities responded to MAM. Participants explained that the person who delivers health messaging shapes the authenticity of information and medicines. Timing and quality of information that was provided was pivotal to programme success or failure. For example, if information is only provided to community leaders the day of or immediately prior to the programme, they are unable to adequately sensitise their community members. In addition, where information was rushed, incomplete or lacking in content, a few CDDs and teachers described that community leaders struggled to share information that would instigate behaviour change and promote medicine acceptance.

*‘The community accepts the medicines although there are usually not enough posters and not enough time during implementation for sensitisation in the markets places*, *churches and mosques’*

*(Participatory meeting with FLHF*, *Ikara LGA*, *Kaduna State)*

*‘Unfortunately*, *the level of information about this program…has reduced and that is why we have serious challenges with people accepting the medicines as compared to the previous year’*

*(Participatory meeting with CDDs*, *Ijebu East LGA*, *Ogun State)*

Just as community leaders can be an asset to the programme, without adequate engagement they can also be a hindrance. For example, if as gatekeepers they are not well informed or do not have a positive perception of the programme, some FLHF Staff described that they would share messages that may result in community members not accepting medicines.

‘Where community leaders are not aware of the medicine distribution they won’t accept it and would also tell their community members not to take the medicines’

*(Participatory meeting with FLHF*, *Abeokuta LGA*, *Ogun State)*

In areas less familiar with CDI approaches, or where new community leaders had emerged, FLHF staff described increasing challenges in the engagement of community leaders due to an expectation of the provision of incentives in exchange for their co-operation. They described this as being due to the initiation of other community health initiatives, such as the polio programme who were providing such incentives.

#### The fundamental role of the health workforce in community mobilisation and sensitisation: what happens when they get tired?

State NTD implementers and frontline health facility staff mainly reported the need for their continuous engagement with the community through sensitisation meetings, particularly prior to MAM. All FLHF staff reported that this helped cross-community information sharing, where participants from different communities were often present in each other’s meetings, resulting in them taking messages back to their own community in advance of their community meeting. CDDs were described to play a critical role in the continuous engagement process, particularly when they had been selected by the community.

*‘The community accepts the drugs during the implementation and this is because they have been sensitized*. *During the exercise prior to our arriving at the next community the people in the previous community have informed each other and will rush to where we are so that they can get the drug’*

***(Participatory Meeting FLHF Staff*, *Ikara*, *Kaduna)***.

FLHF staff and CDDs described CDDs as the connection point between formal health staff and communities, thus taking on the dual role of a community gatekeeper and forming a crucial part of programme delivery. In areas where CDDs had been selected in the ‘traditional’ CDI manner through the use of participatory community meetings, most CDDs and FLHF staff described that community members were accepting of the medicines, mainly because they trusted the CDD. However, some CDDs did describe being challenged or facing criticism by communities, with some community members describing them as ‘*jobless people who go about distributing medicines’ (****Participatory meeting*, *CDDs*, *Yewa North*, *Ogun State)***.

Despite ongoing dedication of health workers and CDDs, in areas where the NTD programme has been implemented for many years (sometimes over 27), community and staff programme fatigue were described by key informants as having led to a complacency regarding community sensitization. In addition, FLHF staff, teachers and CDDs also reported sometimes leaving out ‘geographically hard to reach areas’ due to inadequate support and programme resources, specifically transportation that resulted in them frequently having to use their own resources. Consequently, acceptance of the programme in these areas was compromised, particularly where praziquantel has been integrated with other programmes without an appropriate level of community mobilisation around side effects and their management.

*‘I (stammers) have put in a letter*, *I want to leave this NTD program because I’m tired*, *it’s my money I’m using to go round the communities*, *money which I’m supposed to use and feed my family I go round the Local Government*, *you know my Local Government is one of the largest local Governments in Kaduna so I’m tired of the lack of response*. *When you go to them (government) they will say sacrifice’*

***(KII*, *LGA Level*, *Kachia*, *Kaduna)***.

### Syncretic belief systems and mis-information on medicines

Across both states, religious and traditional beliefs were thought to present a challenge to programme acceptance. For example, health workers in Kaduna and teachers in Ogun described that programme acceptance was particularly low where community members believed in prayer or the use of herbs to prevent disease (particularly when they were attributed to non-medical causes). In Kaduna, health workers described this as particularly pronounced where traditional healers were not engaged in sensitisation or programme delivery.

*‘In my opinion*, *not all of them (community members) accepted us*, *they don’t believe in orthodox medicine*, *they will rather take herbs and concoctions than take the medicines we offer them even though it’s for free’*

**(Participatory Meeting with CDDs, Ijebu East, Ogun)**.

Beliefs about the purpose and origin of medicines also shaped how communities engaged or interacted with MAM. For example, in Kaduna State, particularly in the northern LGAs, most teachers, State and LGA key informants, reported that medicines were often thought to be linked to family planning and would either prevent pregnancy or make men impotent.

*‘Rejection of medicines was due to side effects such as vomiting*, *dizziness*, *big size of the medicines and the fact that some people believe that it is for family planning’*

*(Participatory Meeting with Teachers*, *Igabi*, *Kaduna)*.

The fact that medicines were free had differing impacts on community engagement with the programme. For example, in Kaduna State, some frontline health facility staff and most CDDs described that the medicines being free often meant that community members felt the medicines were being distributed with ulterior motives, they were not good, or not genuine and as a result they refused to swallow the medicines, thereby requiring additional sensitisation.

‘Some would say that any free drug is not a good drug as far as they are concerned’

***(Participatory Meeting with FLHF Staff*, *Ikara*, *Kaduna)***.

In Ogun State however, the fact that medicines were free often meant increased levels of acceptance:

‘Another success to the community is that the medicines are free of charge, and they are happy that government remembers them’

***(Participatory Meeting with FLHF staff*, *Yewa North*, *Ogun)***.

### Side effects and medicine benefits

Side-effects (mainly vomiting and dizziness) and in some rare cases severe adverse events (SAEs) were reported by all participants as a factor that decreased acceptability of MAM and led to high levels of refusals. National key informants described that despite best efforts to sensitise communities using ‘radio jingles’, non-participation in the programme due to side effects was still a major issue.

*‘At times*, *those that have drug reactions once they take it they won’t come back complaining that ‘it itches me’*, *‘my body is swelling’*, *‘it is causing eruptions’ they will not come back they won’t use the drug again it will be abandoned’*

*(****Participatory Meeting with frontline health facility staff Abeokuta LGA Ogun)***.

In Kaduna, most teachers described that pupils tended to experience side effects because of not having eaten prior to taking the medicines. To overcome rejection of medicines due to side effects, some LGA and State key informants described that they had initiated additional training for head teachers and other teachers, particularly around praziquantel, so that they were better equipped for sensitisation and the management of side effects.

Some state key informants also described encouraging better engagement with the frontline health facility during school-based deworming campaigns, either through the referral of pupils to the facility when experiencing side effects, or by asking the health worker to be present during the distribution of the drug to monitor the pupils for side effects. These additional steps had enabled increased acceptability of the medicines by some parents and communities.

Additional reported benefits in physical health such as children passing out worms, often allowed community members to link MAM to educational messages therefore promoting programme acceptance. These improvements in physical health (such as less complaints of stomach ache and passing blood in urine) were viewed as contributing toward increased school enrolment, attendance and performance.

*‘It also helps to prevent worms*. *They also result in good performance of the pupils and increased turn out of students*. *This is because parents were encouraged to send their children to school just for the sake of taking these medicines’*

***(Participatory Meetings with Teachers*, *Ikara*, *Kaduna)***.

### Adaptability

Within the following sub-themes, we present illustrative examples of how key changes in socio-political circumstance (e.g. urbanisation) and social-power relations (e.g. gender) require adaptation to existing programme delivery approaches to ensure equity in programme access.

#### Urbanisation and insecurity

Key informants described that sensitisation can be challenging in urban and peri-urban areas where community leaders have less influence and control due to increased heterogeneity and less collective opinion. Some key informants reported trying to adapt to overcome this through advice from leaders; suggestions included greater sensitisation at communal gathering points and more targeted sensitisation in some areas. This challenge was further exacerbated in areas of political insecurity, particularly when FLHF staff could not access these areas to engage with community leaders. CDDs did not necessarily see this as their role and so medicines are distributed without engaging leaders potentially reducing acceptance and compromising implementation. In security compromised areas, key informants also described engaging these leaders for monitoring activities.

*‘You see the communities we have in this Local Government within the past 2 years*, *we had to revisit…*.*because there is insecurity*, *kidnapping*, *with Fulani attacking the communities especially in the western part of the Local Government making the community leaders in those communities to help monitor activities*.*’*

*(****KII LGA Staff*, *Kachia*, *Kaduna)***.

### Shifting community priorities: Knowledge of visible morbidities and medicine delivery

Where ivermectin had been distributed for a long time, there was often a high level of demand for the programme. Key informants at the State and LGA level and some CDDs linked this demand to the visible morbidity caused by these diseases, either in existence in the community or as depicted in IEC materials used during advocacy visits. In Ogun state, informants and CDDs particularly linked this to onchocerciasis related blindness and a loss of livelihood activities or fear of starvation due to no longer being able to farm.

*‘In some cases*, *in which the affected person with the disease is a farmer*, *he would not be able to do farming activities anymore or even if he can still manage to farm he will not be able to do much*. *Or in the case of someone with river blindness*, *he will not be able to do any work*. *He will just be at home*, *starving which is why they (community members) are asking for the medicine’*

***(Participatory Meeting with CDDs*, *Ijebu East*, *Ogun)***.

Despite these perceived benefits, in Kaduna State teachers and CDDs reported that there are increasing challenges with people refusing to take medicines due to a lack of perceived need (i.e. they are not sick) or a lack of visible morbidity. They described this as being exacerbated in areas where NTD morbidity prevalence or burden had been reduced (a key success of the NTD programme), with some key informants at the federal level describing that some communities feel that the programme has worked and is therefore no longer necessary.

‘Some elderly people refuse to take the medicines because they feel they don’t need them as they are healthy without taking the drug’

***(Participatory Meeting with CDDs*, *Kachia*, *Kaduna)***.

‘*Sometimes we come across people that reject the medicines because they don’t understand the importance of the medicines as they believe that there is nothing wrong with them physically*’

***(Participatory meeting FLHF staff*, *Ikara LGA*, *Kaduna State)***.

Some CDDs and FLHF staff in Ogun and Kaduna perceived that communities were more reluctant to accept MAM and engage with the NTD programme when their other development needs were not being addressed. They described higher levels of medicine refusal where communities felt that they had competing community priorities such as a need for better infrastructure, provision of food, safe drinking water and fertilisers. Poverty was also frequently cited as the main concern of the target communities.

*‘if we get to the community some of the elders will say they are hungry and we are giving them medicines do we want hunger to kill them*? *Do we want them to use the drug on an empty stomach*?*’*

***(Participatory meeting with CDDs*, *Abeokuta North LGA*, *Ogun State)***.

#### Migrant groups

In Kaduna State, LGA officers reported that within migrant populations, such as Fulani, failure to adapt sensitisation processes to align to their traditional cultural practices can compromise the success of the programme. For example, not eating food provided by the Fulani prior to offering them the medicines will result in instant refusal. The use of measuring sticks for distribution will also lead to refusals as sticks are used to measure corpses. Key informants described enhancing training for CDDs to address these issues but also explained that more needs to be done to ensure their continued engagement with the programme.

‘*In some communities they don’t allow us to use sticks in measuring their height because that is how they usually take measurement of the dead for burial arrangements and hence the belief that if they are measured they will die soon*’

*(Participatory Meeting with CDDs*, *Kachia*, *Kaduna)*

#### Cultural and language variation

In Ogun State, some FLHF staff also described distribution challenges based on cultural variations. In some communities where there are groups from multiple tribal backgrounds, community members from the ‘non-host’ tribe would refuse medicines as they thought that they were designed to harm them. This was particularly common in boarder areas and FLHF staff described having to enhance sensitisation activities to these populations and in other areas where language was not shared. In instances where health workers and community members had no common dialect, this often resulted in medicine refusal.

*‘We talk about challenges which are language barriers*. *In some of our communities we have Hausa*, *Yoruba*, *Cotonou are there*. *I hear a kind of Ijebu*, *when we want to talk to them that they should use the drug*. *We don’t understand each other*. *It is a barrier and it’s difficult to convince them*. *So*, *we will look for intermediary that will help us interpret*. *Religion barrier is there*. *We all know them*, *this people that believe in healing without medicine*. *They will collect it but keep it down and some will reject it’*

***(Participatory Meeting with FLHF*, *Ijebu East*, *Ogun)***.

‘*There is a complete ward that is sometimes left out because it is hard-to-reach X ward because of distance and terrain. Other places include X and X having distance and insecurity issues*’

*(KII*, *LGA Level*, *Igabi*, *Kaduna)*.

#### Gender roles and livelihoods

Most CDDs reported difficulty in accessing certain households especially where for religious reasons male CDDs had limited or no access to female members of the household without the permission of the head of the household. This was exacerbated where men worked outside of the community. In communities that were agrarian, CDDs reported difficulty in reaching people who were farmers and hunters during the rainy season. Those who were long distance drivers and had to leave their homes early in the morning also posed a challenge as shortened distribution times didn’t allow for more time to keep going back to these houses.

*‘more males and females should participate in the program because the male CDDs are not allowed to enter some houses in such cases it*’*s the females that are allowed in to administer medicines in that particular family*, *so we suggest that more females should be involved’*

*(Participatory Meeting with FLHF*, *Ikara*, *Kaduna)*

*‘Why people are missed is because some will go to the farm and will not come back on time*. *When you get to their houses you might not find them at home*.

*(Participatory Meeting with FLHF*, *Abeokuta North*, *Ogun)*

## Discussion

Overall, our results indicate that generally, CDI approaches have provided an acceptable platform for reaching at-risk communities with preventive health services (particularly MAM). However, there are still some adaptations that could be made to programme delivery to increase and sustain programme equity.

**Fig 1:**
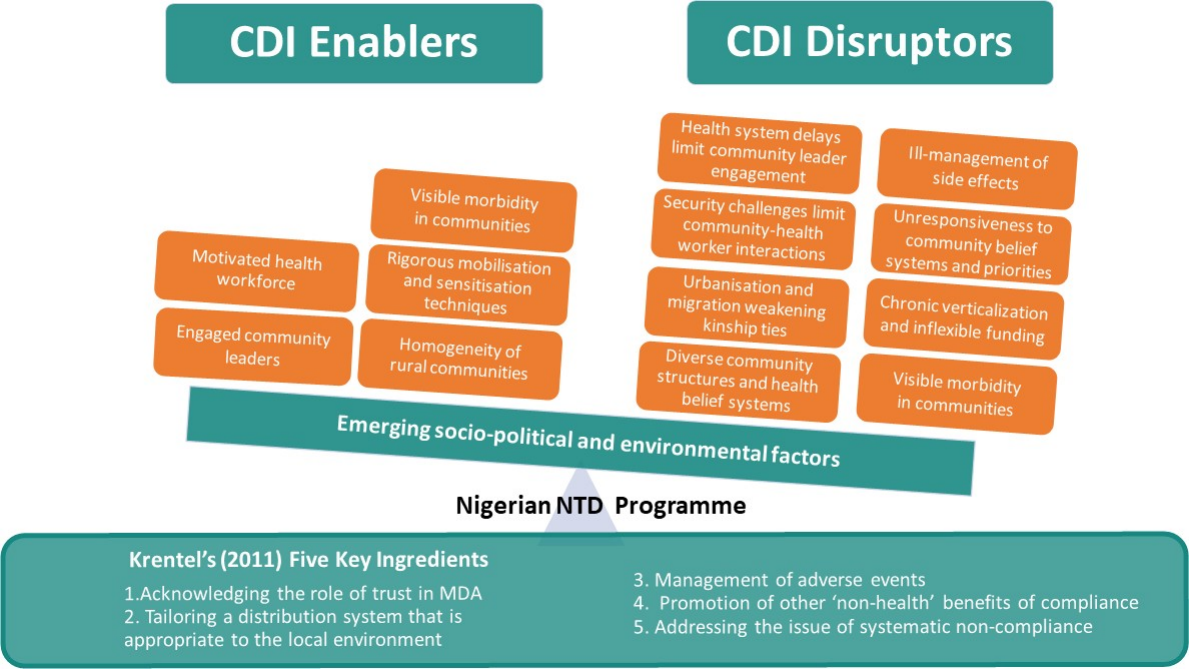
Engaging and disrupting factors for a successful community engagement program.

In their 2013 review, Krentel *et al*, identify ‘five key ingredients’ that are essential in shaping individual acceptance of MAM, with specific relation to LF. These ingredients include: 1) Acknowledging the role of trust in MDA; 2) tailoring a distribution system that is appropriate to the local environment; 3) management of adverse events; 4) promotion of other ‘non-health’ benefits of compliance; and 5) addressing the issue of systematic non-compliance [14]. CDI approaches have been frequently described as the best way to achieve these ‘key ingredients’ and factors that have contributed to the success of the approach have been largely documented in the literature and reflected in this study are; homogeneity of rural communities, rigorous mobilisation and sensitisation techniques supported by the presence of visible morbidity in communities, motivated and engaged community leaders and health workers [14] (see Fig 1). However, factors that disrupt the CDI model and ability of MAM to achieve these ‘five key ingredients’ are less documented. Fig 1 below illustrates key disrupting factors to the attainment of these ‘five key ingredients’ emerging from our analysis of existing CDI approaches for MAM in Kaduna and Ogun state. Our discussion is structured around each of these ‘key ingredients’ where we explore how changes in context can present challenges to CDI approaches in their current form.

### Challenges in establishing trust in alternative contexts and maximising the use of social capital

Community directed approaches as the key delivery platform for MAM have relied heavily on the homogenous nature of rural communities to sustain engagement. Sensitisation and awareness activities through appropriately chosen community leaders and structures which build on social capital and trust have been of great benefit to both community and school-based MAM interventions [14,15]. Our results reinforce this highlighting that the structure of typical rural communities in Nigeria, underpinned by strong kinship ties [16,17], has been of historical benefit to the implementation of the NTD programme in establishing trust and consequent acceptance of medicine delivery. However, urbanisation, migration and an increase in presence of security compromised areas in Nigeria has continued to have an influence on the socio-cultural landscape, with community structures and belief systems adapting, becoming more diverse and in many areas family and kinship ties weakening [18]. This has presented a challenge for the programme in establishing and maintaining trust in less homogenous settings such as those that are more urban or experiencing periods of instability [15]. Understanding who and what structures to engage with in alternate contexts where community leaders or organisations have less control over collective opinion is therefore critical for the NTD programme in establishing trust and in ensuring medicine acceptance. This requires new partnerships that reflect different contexts and is essential to ensure no one is left behind in programme delivery.

Molyneux (2018) identified socio-political upheavals such as conflict, disease outbreak and or environmental impacts as a challenge to achieving NTD elimination targets, stating a need for innovative approaches to program delivery [10]. Our results show that face to face interactions with health workers and CDDs are essential in establishing trust and rapport with communities and their leaders [14]. Where access to communities for health workers was limited based on security issues, challenges in establishing trust were exacerbated. Our results highlight that reliance on tacit knowledge and engagement of residents (particularly community leaders) and CDDs from security compromised areas is therefore critical in the use and development of tailored approaches that ensure these areas are reached. Consequently, as suggested by Gonzales et al (2019), our results support the idea that, when leveraged effectively social capital (in the form of community leaders and others) has the potential to not only increase programme acceptance but also support the design, delivery and monitoring of MAM and other health interventions [15].

### Tailoring distribution strategies to the local environment: Programmatic challenges and a need to adapt to the cultural context

As emphasised above, community leaders are an invaluable resource for MAM programme delivery and programmes working with leaders have a greater chance of success [14]. As found in other contexts [14], challenges with the quality and timing of information shared to community leaders limits their sustained engagement and affects their ability to share information effectively with communities, ultimately compromising programme acceptance. Reasons identified for inadequate engagement with community leaders were three-fold and normally attributable to programme delivery challenges including: 1) delays in the drug supply chain, meaning little time between training distribution and reporting, rendering CDDs unable to adapt and cascade information messaging; 2) health worker fatigue and insufficient resources to deliver appropriate sensitisation, resulting in substantial out-of-pocket expenditure by frontline health personnel; and 3) an influx of other health programmes that provide incentives for community leader engagement (e.g. polio) leading to dis-engagement from many leaders when the MAM programme is unable to match such incentives. Programmatic factors that compromise community directed approaches are not uncommon but require innovation and adaptation [19]. Solutions to some of these programmatic challenges in other sub-Saharan African contexts have included: standardisation of incentive structures across all disease programmes; and provision of monthly stipends and logistical supplies to community health work cadres [20]. However, most of these solutions are critically dependent on the ability of governments to adapt funding and implementation structures to financially and logistically contribute toward supporting such efforts. Donors and programme implementing partners should also be more flexible and responsive by instigating less restrictive reporting and funding structures. This will likely contribute toward enabling programmes to respond to their own challenges and implement as they desire, ultimately moving away from chronic verticalization [21] in the delivery of MAM to programmes that allow more flexible planning and implementation at the local level.

MAM delivery currently relies on the roll out of a “one size fits all” approach to medicine distribution. Whilst this has achieved many successes, as Parker and Allen suggest; ‘roll out of a single policy in a uniform way is unlikely to be effective, inevitably it will be influenced by the political, economic, historic and social context in which it occurs’[22]. This is exacerbated as standardised approaches also move away from the true roots of the CDI model that rely on communities’ ownership [7]. Adapting distribution strategies to be responsive to the local socio-cultural and programme delivery context is therefore essential in minimising an overreliance on top-down vertical approaches [21] and is critical to achieving equity in programme delivery, even where overarching programme goals may already be achieved. There are key sub-populations such as Fulani migrants where standardised elements of MAM delivery including measurement using a stick are culturally unacceptable and challenge programme acceptability. In areas where there are mixed tribal groupings, we also found that who is selected as the CDD is of critical importance as notions of belonging and language matters [23]. Small programme adaptations, such as taking a different approach to measurement, selecting multiple CDDs from different social backgrounds and genders from within one community, or ensuring the modification of materials to meet different communication needs would likely enhance programme equity [23].

The same was also true regarding gender norms, traditional and religious beliefs and their interactions with programme acceptability. Syncretic belief systems that see the convergence of ‘medical’ and ‘traditional’ understandings of medicines and disease often shape the acceptance of ‘westernised’ medicine or ‘formal’ health interventions in different ways in different settings [24]. Some communities were described as refusing to accept medicines due to the belief that more ‘traditional’ medicines were required to treat targeted diseases. Additionally, in Ogun, medicines being donated for free pleased communities and made them feel of value to the government, whilst in Kaduna, a convergence of religious and traditional belief systems led to communities believing that medicines which are donated for free restrict fertility and should not be trusted or accepted. Gendered norms also restricted access to some individuals in communities, particularly in Kaduna where male CDDs could not enter certain households without permission of the household head, creating the potential for some women to remain ‘invisible’ to the NTD programme when not treated or accounted for within programme records [23]. There is a clear need for NTD programmes to develop sensitisation and awareness activities that can respond to gendered norms and syncretic belief systems within target communities to allow individuals to make appropriately informed choices about their willingness to accept MAM [22]. Failure to do so can compromise the trust that communities have in MAM and other health interventions. Cumulatively, all these factors emphasise that adaptability to context is of critical importance in implementing MAM in this setting [3].

### Management of adverse events and the introduction of new medicines

Across many contexts, experience of, and rumours about side effects and in some cases extreme adverse events are some of the most documented challenges in ensuring the acceptability of MAM [14]. Results in this study supported these findings indicating that side effects, mainly vomiting and dizziness, were described by informants as decreasing the acceptability of MAM leading to high levels of refusals. Side effects tended to be identified in relation to the distribution of praziquantel, particularly in the initial years of MAM when disease burden is highest [14,25]. Failure to adequately manage the experience of side effects and adverse events or conduct sensitisation regarding the risk and likelihood of certain types of side effects is known to and as supported here have negative impact on programme outcomes [26]. Within the context of integrated programme delivery, it is critical that these issues are addressed to ensure that experiences and rumours regarding side effects in relation to one medicine, in this case praziquantel, do not damage the long-standing trust or acceptance communities have regarding the acceptance of other medicines (e.g. ivermectin) delivered through the same or similar platforms. Best practices identified to ensure the effective management of side effects are: intensive advocacy with community leaders; immediate action in response to side-effects using pharmacovigilance guidelines; additional and targeted training with community volunteers and health workers; and in the case of school-based deworming, strengthening cross-sectoral collaboration so that health workers are present during distribution. In prioritising the integration of other diseases within strong existing community platforms, it is critical to remember the impact and consequence this can have on community understandings, the complexities it adds to health messaging, and the additional support needs of community volunteers and health workers to be able to manage integration efforts at the frontline [27].

### Morbidity and changing priorities: A need to think beyond direct health benefits

Sensitization efforts linked to MAM frequently emphasize the health benefits of a ‘treatment’ or ‘cure’ for targeted diseases. However, the steady decline of visible morbidity associated with NTDs (a key programme success) combined with traditional beliefs about causes and treatment has led to a low perceived need for MAM in some communities. Ultimately, as Krentel et al (2013) describe ‘it is hard to convince people who feel well to take medicines’. ‘Wellness’ in this case is intrinsically linked to an individuals’ interpretation of ‘ill-health’ and not directly linked to biomedical understandings of disease [24]. Although not true in all areas, with some still experiencing current or memories of physical morbidities linked to onchocerciasis, there is a need for the NTD programme to be able to adapt sensitization messaging to focus on more than just the ‘treatment’ or ‘cure’ of target diseases. Other non-health benefits identified by respondents in our study that could be capitalized in more diverse sensitization activities are increased school attendance, reduction in lice and an increase in libido, and the use of patient advocates. Without significant adaptation to sensitization messaging, our results indicate that despite short-term acceptance in areas where high levels of visible morbidity are experienced, sustained engagement with MAM interventions is likely to be compromised.

Finally, our results also show that changing and wider development priorities amongst community members also shaped program acceptance. Preferences for food and infrastructure (such as roads and bridges) were prioritised by community members who disengaged with the programme as they could not understand why this programme would continue when they had greater needs in other areas. The links between NTDs and the broader environment are well established, and wider social determinants including agricultural infrastructure, irrigation systems and improved sanitation are critical in striving toward elimination of these diseases [28,29]. There is, therefore, a unique and essential opportunity for NTD programmes to be able to partner across sectors to address structural determinants of NTD risk whilst responding to community identified development priorities that may also contribute to enhanced medicine uptake.

### Addressing the issue of persistent refusals: A missing link for programme implementers?

Persistent refusal was not raised as a key issue by programme implementers. However, some of the key issues raised above including the impact of side effects, challenges in creating trust and rapport with communities, and a lack of perceived need of medicines, are all likely to contribute toward a persistent dis-engagement with the NTD programme for some individuals. Further work and discussions are therefore necessary with programme implementers at all levels in Nigeria to understand how to identify and engage with persistent refusers to avoid ‘continued sources of infection’[14] within target communities.

### Strengths and weaknesses

A key limitation of our study was the absence of the ‘voices’ of community members. However, this study was designed to serve as a context analysis for latter pieces of work and we felt that the engagement of multiple groups of CDDs who serve as a link point between communities and the health system would support in mitigating this challenge. A key strength of our research was being able to engage with implementers from varying levels of the health system using participatory and in-depth methods that allowed these actors to express their views and concerns in their own way and based on their own priorities. Many implementers reflected throughout data collection on how this was rare for them despite having been engaged in multiple research studies and working for the NTD programme for many years. Finally, the collation of data from across two very diverse states in Nigeria contributes towards the generalisability of our findings, however in future work, the inclusion of states from all geo-political zones based on contextual variability would further enhance this studies generalisability.

## Conclusion

Nigeria has Africa’s largest NTD burden and has achieved a lot of successes to date utilising the CDI model in the MAM. Meeting the 2020 targets and achieving the ‘end game’ in changing contexts will require additional efforts and strategies. Understanding factors that currently disrupt the CDI model, presented in this paper, will be critical. Addressing these factors means supporting frontline providers with the time, tools, resources and supervisory structures to enable them to build strategic and trusting relationships with fluid and changing communities in different contexts thereby ensuring no one is left behind.
